# New record of an estuarine polychaete, *Neanthesglandicincta* (Annelida, Nereididae) on the eastern coast of Peninsular Malaysia

**DOI:** 10.3897/zookeys.831.28588

**Published:** 2019-03-18

**Authors:** Nur Fazne Ibrahim, Yusof Shuaib Ibrahim, Masanori Sato

**Affiliations:** 1 School of Marine and Environmental Sciences, Universiti Malaysia Terengganu, 21030, Kuala Nerus, Terengganu, Malaysia Universiti Malaysia Terengganu Terengganu Malaysia; 2 Department of Earth and Environmental Sciences, Graduate School of Engineering and Science, Kagoshima University, 1-21-35 Korimoto, Kagoshima 890-0065, Japan Kagoshima University Kagoshima Japan

**Keywords:** Kuala Ibai, paragnath, polychaete, Setiu Lagoon, South China Sea, taxonomy, Tumpat

## Abstract

An estuarine species of Nereididae (Annelida), *Neanthesglandicincta* (Southern, 1921) has been newly recorded on the eastern coast of Peninsular Malaysia located in the South China Sea based on 23 specimens collected from three estuaries (Tumpat, Kelantan Delta, Kelantan; Setiu Lagoon, Terengganu; Kuala Ibai, Terengganu). The morphological characteristics of the Malaysian specimens agree well with those of the previous original description and the redescription of *N.glandicincta* based on Indian, Myanmar and Singapore specimens. The number of paragnaths in all groups on the proboscis of our Malaysian specimens is within the range of the intraspecific variation of *N.glandicincta* as shown in the previous descriptions. An identification key to species of the *Neanthesglandicincta* species complex, which includes two morphologically similar species, is provided.

## Introduction

Estuaries are ecologically important habitats which serve as critical transition zones between freshwater and marine habitats (Levin et al. 2001). In general, nereidid polychaetes often occur as major components of the macrobenthic fauna in estuaries and play important roles in the nutrient cycling of an estuary ecosystem (Sato 2017). However, taxonomic knowledge of nereidid fauna in tropical Asia seems to be insufficient although the area has the greatest diversity of coastal species in the world (Tittensor et al. 2010). In Malaysia, only six nereidid species belonging to three genera (*Namalycastisrhodochorde*, N.cf.abiuma, *Namalycastis* sp., *Dendronereides* sp., Perinereiscf.nuntia and *P.aibuhitensis*) have been recorded with a published taxonomic account (Idris et al. 2012, Ibrahim et al. 2017), while approximately 700 species belonging to 45 genera have been recorded worldwide (Santos et al. 2005, Read and Glasby 2015).

In Asian tropical estuaries, two nereidid species, *Neanthesglandicincta* (Southern, 1921) and *Composetiaburmensis* (Monro, 1937), have been most commonly reported (Lee and Glasby 2015). Lee and Glasby (2015) demonstrated that *C.burmensis* is a junior synonym of *N.glandicincta*, and newly found a cryptic species from eastern Singapore, which is morphologically very similar to but distinct from *N.glandicincta*, and described as *Neantheswilsonchani*.

During our survey of the nereidid fauna in estuaries located on the eastern coast of Peninsular Malaysia, we found *N.glandicincta*, which commonly occurs in all of the three sites surveyed, without any occurrence of *N.wilsonchani* in spite of geographical proximity between our sampling sites and the type locality of *N.wilsonchani*. In the present paper, we describe *N.glandicincta* as a new record from the eastern coast of Peninsular Malaysia.

## Materials and methods

**Figure 1.**
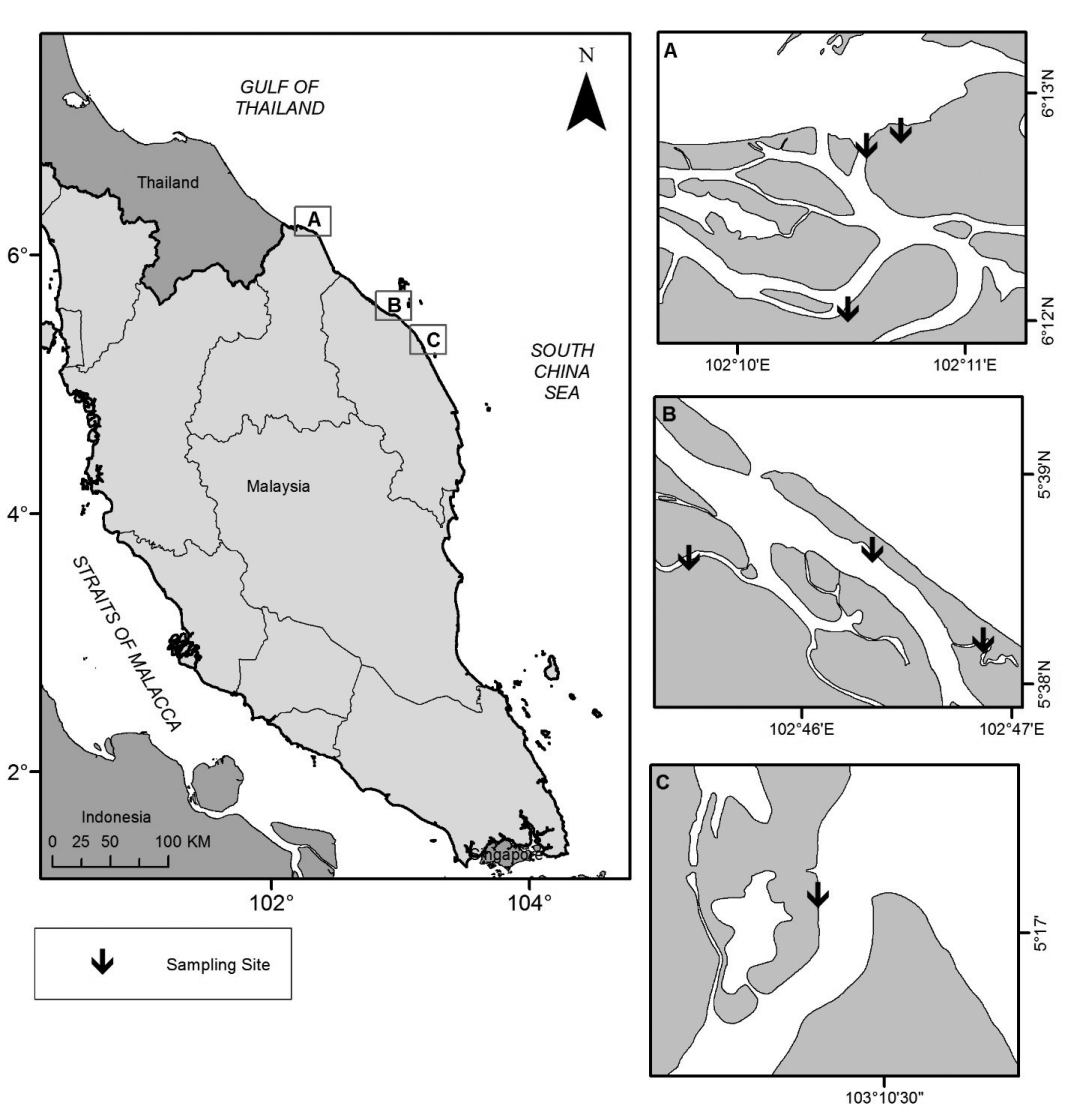
Map showing three estuaries in the eastern coast of Peninsular Malaysia. **A** Tumpat, Kelantan Delta, Kelantan **B** Setiu Lagoon, Terengganu **C** Kuala Ibai, Terengganu.

Field sampling for nereidid specimens was carried out at three estuaries located on the eastern coast of Peninsular Malaysia (Fig. 1): Tumpat, Kelantan Delta, Kelantan (Fig. 2A) in August 2009 (three sites); Setiu Lagoon, Terengganu (Fig. 2B, C) in August 2009 (two sites) and 2015 (one site); and Sungai Ibai, Kuala Ibai, Terengganu (Fig. 2D) in August 2009 (one site).

**Figure 2.**
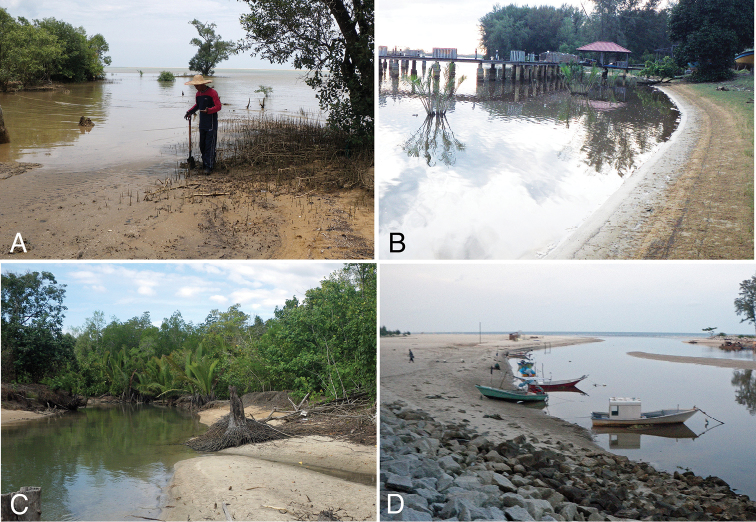
Sampling sites in three estuaries in the eastern coast of Peninsular Malaysia. **A** around river mouth of Sungai Mak Neralang in Tumpat, Kelantan Delta, Kelantan (photographed on 13 August 2009) **B** site 102 at the eastern coast of Setiu Lagoon, Terengganu (photographed on 10 August 2009) **C** muara Kuala Setiu at the western coast of Setiu Lagoon, Terengganu (photographed on 6 August 2015) **D** around river mouth of Sungai Ibai (photographed on 12 August 2009).

Specimens were collected from sediment samples dug out from intertidal bottoms using shovels, fixed in 80% ethanol, and transferred to fresh 80% ethanol for preservation. The salinity of the surface or interstitial water (water kept in a hole dug in the sediment surface at low tide) was measured using a SCT meter (Quanta Multi-Parameter Probe).

For preserved specimens, the anterior maximum body width, excluding parapodia (BW) was measured at a scale of 1-mm units; for complete specimens, its body length from the anterior end of the prostomium to the posterior end of the pygidium, excluding the anal cirri (BL) was measured, and the number of total chaetigers was also counted. The paragnaths in each group on the proboscis were counted under a stereoscopic microscope both the right and left sides of Groups II and IV were counted but only the larger count was reported. Photographs of the specimens were taken using a digital camera (AM4023X Dino Eye) attached to stereoscopic (Olympus SZX7) and compound (Leica DM300) microscopes. Drawings were prepared using a camera lucida attached to the microscopes. A map drawing was prepared using the ArcGIS 10.3 software.

The usage of terminology of paragnath groups on the proboscis, and parapodial and chaetal morphology is according to Bakken and Wilson (2005).

Specimens were deposited in the South China Sea Repository and Reference Center of Universiti Malaysia Terengganu (**UMT**) and the National Museum of Nature and Science, Tsukuba, Japan (**NSMT**).

## Systematics

### Family Nereididae Blainville, 1818

#### Genus Neanthes

Taxon classificationAnimaliaPhyllodocidaNereididae

Kinberg, 1865

Neanthes Kinberg, 1865: 171–172; Imajima 1972: 102; Fauchald 1977: 89; Wu et al. 1985: 143; Khlebovich 1996: 102; Bakken 2002: 328; Bakken and Wilson 2005: 527; Glasby et al. 2011: 363; Sato 2013: 35; Bakken et al. 2018: 29.

##### Type species.

*Neanthesvaalii* Kinberg, 1865.

##### Diagnosis.

Prostomium with entire anterior margin, one pair of antennae, one pair of palps. Eyes present or absent. Eversible proboscis usually with conical paragnaths on both maxillary and oral rings; paragnaths on oral ring occasionally degenerating to minute ones or completely lost; paragnaths occasionally emerging from plate-like basement; smooth bar-like paragnaths present or absent on group IV of maxillary ring. Four pairs of tentacular cirri. Parapodia biramous, except first two pairs; notoaciculae present or absent on chaetigers 1 and 2. Dorsal cirrus lacking basal cirrophore. Notochaetae homogomph spinigers. Upper neurochaetae including homogomph spinigers and heterogomph falcigers; heterogomph spinigers present or absent. Lower neurochaetae including heterogomph falcigers; homogomph and heterogomph spinigers present or absent. Neuropodial heterogomph falcigers occasionally with varying degrees of fusion of chaetal shaft and blade in posterior body.

##### Remarks.

*Neanthes* is a large genus, considered to be polyphyletic (Bakken and Wilson 2005; Glasby et al. 2011). The generic diagnosis is modified here from Sato (2013) to allow for the occasional absence of paragnaths on the oral ring of the proboscis of the *Neanthesglandicincta* species complex (see below).

#### *Neanthesglandicincta* species complex

**Diagnosis.** Conical paragnaths present in all of groups I, II, III, and IV on maxillary ring of proboscis. Only few minute rudimentary paragnaths or none present in groups VI and VII–VIII on oral ring of proboscis; paragnaths absent in group V; single round papilla usually present in group VI, with single minute paragnath, or no paragnaths, seated on tip of papilla. Uniramous parapodia of first two chaetigers without notoacicula. In following biramous parapodia, notopodia, consisting of dorsal cirrus and three ligules/lobe (dorsal ligule, prechaetal lobe and ventral ligule) throughout. Neuropodia, consisting of four ligules/lobes (superior lobe, inferior lobe, postchaetal lobe, ventral ligule) and ventral cirrus present in anterior and middle body; superior lobe absent in posterior body. Upper neurochaetae includes homogomph spinigers with long blades and heterogomph spinigers with short blades throughout; some or most of heterogomph spinigers replaced by heterogomph falcigers in middle body. Lower neurochaetae include heterogomph spinigers with long blades and heterogomph spinigers with short blades throughout; some or most of heterogomph spinigers with short blades replaced by heterogomph falcigers in middle body. Heterogomph falcigers first appear around chaetiger 20. Conspicuous dark glandular patches present in notopodial dorsal ligules.

**Geographical distribution.** The coast of Indian Ocean (Iran, India, Bangladesh, Myanmar), Singapore, the coast of South China Sea (Peninsular Malaysia, Thailand, Vietnam, China, Taiwan), and eastern Australia. Based on Southern (1921), Monro (1937), Fauvel (1932, 1939, 1953), Khlebovich (1963), Rullier (1965), Wu (1967), Wu et al. (1985), Muir and Maruf Hossain (2014), Lee and Glasby (2015), Bonyadi-Naeini et al. (2017), and the present study.

**Remarks.** Two species, *Neanthesglandicincta* (Southern, 1921) and *N.wilsonchani* (Lee & Glasby, 2015), are included in this species complex at present. The two species are distinguishable only by the numbers of paragnaths (Lee and Glasby 2015; see the key below).

##### Key to species of the *Neanthesglandicincta* species complex

**Table:** 

1	Paragnaths more than 30 in group III, more than 70 in total	*** N. glandicincta ***
–	Paragnaths fewer than 30 in group III, fewer than 50 in total	*** N. wilsonchani ***

##### Neanthes glandicincta

Taxon classificationAnimaliaPhyllodocidaNereididae

Southern, 1921

Figs 3 , 4

Nereis (Nereis) glandicincta Southern, 1921: 589–593, pl. 23, fig. 9A–L, text fig. 5a–c.
Nereis glandicincta : Fauvel 1932: 92–93; Fauvel 1953: 181–182, fig. 91f–h.
Neanthes glandicincta : Lee and Glasby 2015: 80–85, figs 7–9.
Ceratonereis burmensis Monro, 1937: 532–536, fig. 1a–f; Ng et al. 2011, in part.
Nereis (Ceratonereis) burmensis : Fauvel 1953: 196–197, fig. 97d–f.
Ceratonereis (Composetia) burmensis : Hartmann-Schröder 1985: 49 (list); Chan 2009: 165–167, fig. 5a–r, in part.

###### Type locality.

Brackish lakes or pools at four localities in Barantolla, Dhappa and Garia, near Calcutta in India (26 syntypes) (Southern 1921).

###### Material examined.

Tumpat, Kelantan Delta, Kelantan, Malaysia: a specimen (BW, 1.3 mm; UMTAnn 428), around the jetty (6°12'03"N, 102°10'29"E), coll. M. Sato, 13 August 2009; 5 (BW, 1.6–2.0 mm; NSMT 113250), around the river mouth of Sungai Mak Neralang (6°12'46"N, 102°10'34"E), coll. M Sato, 13 August 2009; 2 (BW, 1.5–2.0 mm; UMTAnn 429), natural mangrove forest (6°12'50"N, 102°10'43"E), coll. M Sato, 13 August 2009. Setiu Lagoon, Terengganu, Malaysia: 3 (BW, 1.6–1.8 mm; UMTAnn 430), site 102, northwest of Terrapuri Heritage Village, Penarik (05°38'12.5"N, 102°46'52"E), coll. M Sato et al., 10 August 2009; 8 (BW, 1.5 mm –2.0 mm; NSMT 113251), site 103, northwest of site 102 (05°38'38.4"N, 102°46'20.2"E), coll. M Sato et al., 10 August 2009; 2 (BW, 1.2–1.7 mm; UMTAnn 431), Muara Kuala Setiu (05°40'26.3"N, 102°43'17.4"E), coll. YS Ibrahim et al., 6 August 2015; Kuala Ibai, Terengganu, Malaysia: 2 (BW, 0.7 mm –1.1 mm; UMTAnn 432), around the river mouth of Sungai Ibai (5°17'04"N, 103°10'23"E), coll. M Sato et al., 12 August 2009.

**Figure 3.**
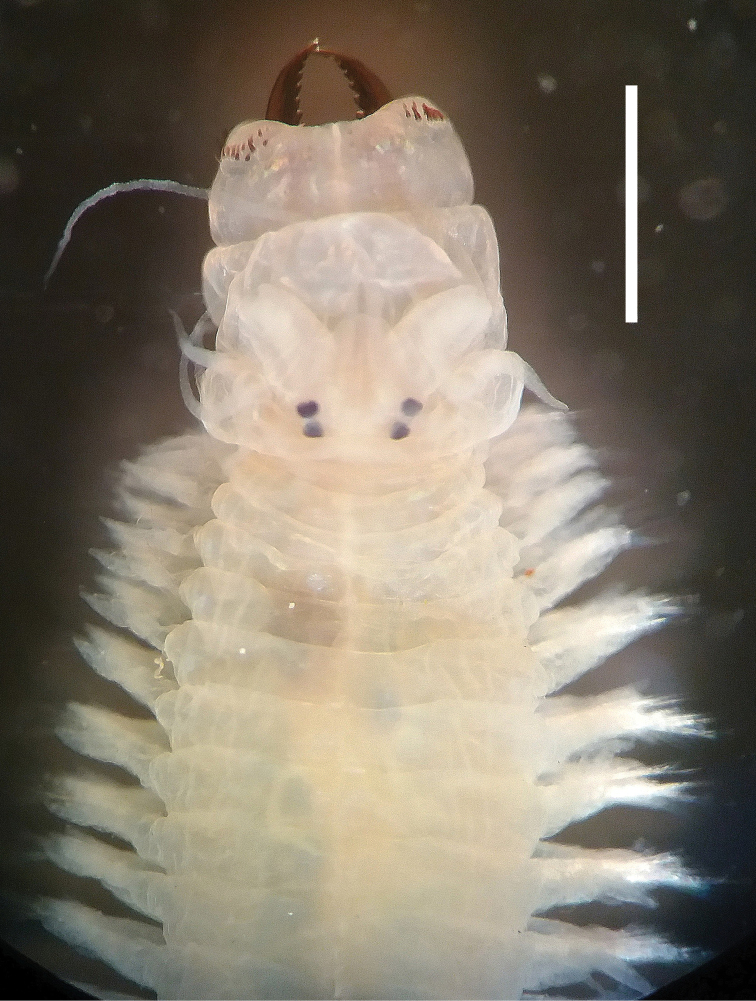
Photographs of a preserved specimen of *Neanthesglandicincta* (Southern, 1921) collected from Setiu Lagoon, Terengganu, Malaysia (UMTAnn 431). **A** dorsal view of the anterior body with everted proboscis **B** ventral view of the anterior body with everted proboscis. Scale bar: 1 mm.

**Figure 4.**
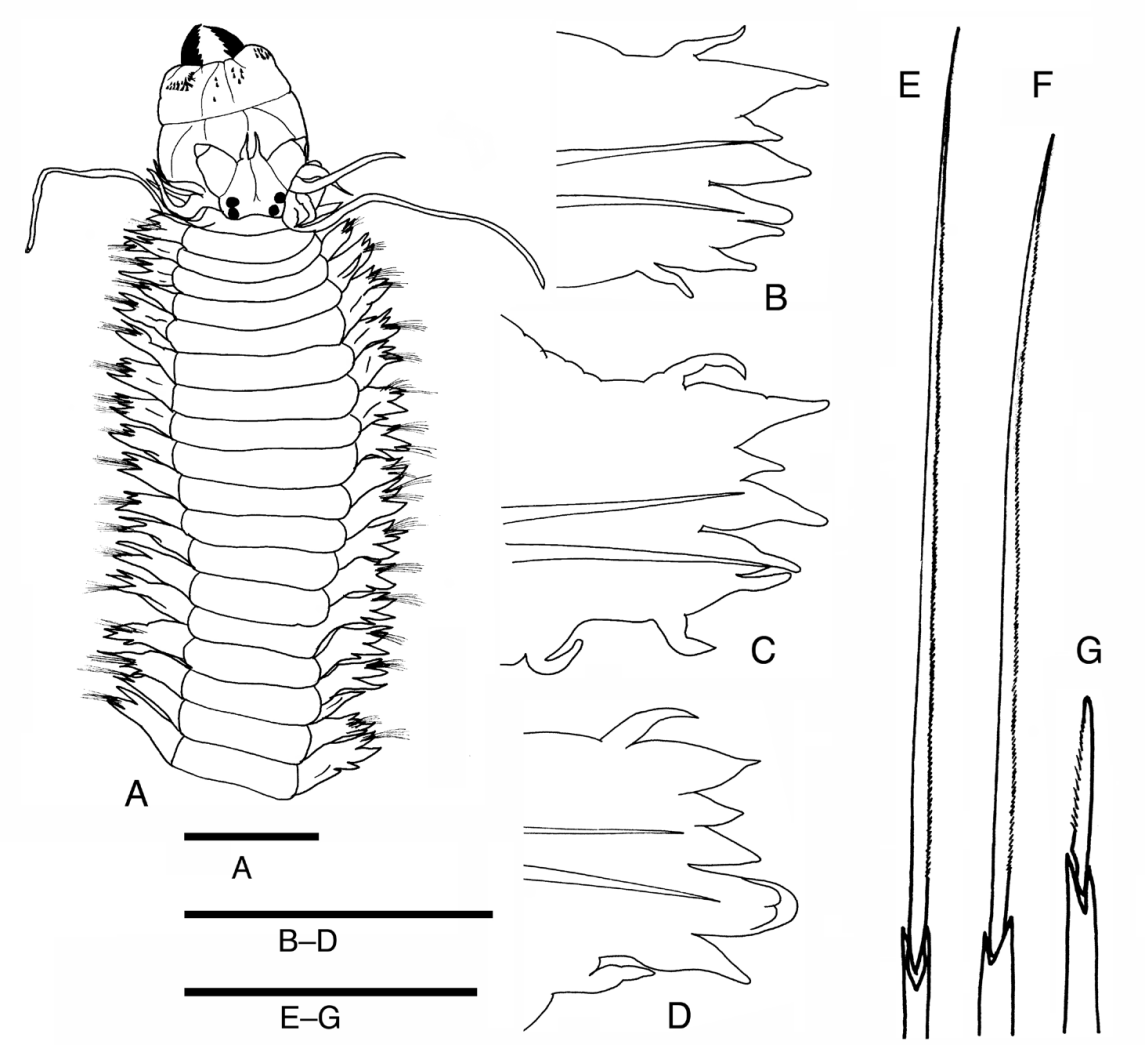
Drawings of a specimen of *Neanthesglandicincta* (Southern, 1921) collected from Setiu Lagoon, Terengganu, Malaysia (UMTAnn 431). **A** dorsal view of the anterior body with everted proboscis **B–D** anterior view of parapodia **B** parapodium 10 **C** parapodium 18 **D** parapodium 35 **E** homogomph spiniger from notochaetae **F** heterogomph spiniger from lower neurochaetae **G** heterogomph falciger from neurochaetae. Scale bars: 1 mm (**A, B–D**); 0.1 mm (**E–G**).

###### Diagnosis.

Based on Southern (1921), Lee and Glasby (2015), and the present study. Maxillary ring of proboscis with conical paragnaths (0–17, usually approx. 10, scattered and unequal in group I; 10–23, large and curved in group II; 30–63, in 4 rows of transversely elongated bands in group III; 10–22, large in group IV). Oral ring of proboscis with only few minute rudimentary paragnaths, often with none (0 in group V; 0 or 1, seated on tip of round papilla in group VI; up to approx. 8 in a single row in Group VII-Group VIII). Total paragnaths more than 70.

###### Description.

Largest complete specimen 70 mm BL, 2.0 mm BW, with 132 chaetigers. Colour in preserved specimens is whitish cream with brownish pigmentation on prostomium, anterior part of palps, and dorsum of anterior chaetigers. Sub-pentagonal prostomium with a pair of smooth tapered antennae situated at anterior end (Figs 3, 4A). A pair of palps with massive palpophores and short conical palpostyles. Two pairs of eyes arranged trapezoidally (anterior pair with space wider than that of posterior pair); anterior pair reniform and slightly larger; posterior pair round and smaller. Mid-longitudinal white slit present on dorsal anterior surface of prostomium. Peristomium with four pairs of tentacular cirri of unequal length; posterodorsal tentacular cirri longest, reaching back to chaetigers 6–12 (Figs 3, 4A).

Proboscis with a pair of light brown jaws, each with approx. ten teeth. Typical conical paragnaths present on maxillary ring (Figs 3, 4A); number of paragnaths on each group are as follows (Table 1): group I: 3–13, scattered and unequal; group II: 13–20 in two arched rows, marked large paragnaths with sharply tapering and curved tip present in middle position; group III: 39–58, in three or four rows of transversely elongated bands; group IV: 11–17 in a triangular patch with markedly large paragnaths present in posterior position. Oral ring sometimes expanded into a trapezoidal shape at full-everted proboscis, with only a few or no rudimentary paragnaths; number of paragnaths on each group are as follows (Table 1): group V: none; group VI: 0 or 1 minute paragnath seated on tip of small round papilla, sometimes only papilla present; groups VII–VIII: 0 or 1 minute paragnath present. Total number of paragnaths 94–137.

**Table 1.** Variation of morphological characteristics of *Neanthesglandicincta* collected from three estuaries in the east coast of Peninsular Malaysia in the present study, in comparison with data from previous studies in other countries.

**Locality**	**Number of specimen(s)examined**	**Body width^2^**	**Body length^2, 3^**	**Number of total**	**Number of paragnaths^4^**							**Total^6^**
**(site no. in the present study)^1^**		**(mm)**	**(mm)**	**chaetigers^2, 3^**	**I**	**II^5^**	**III**	**IV^5^**	**V**	**VI^5^**	**VII–VIII**	
**Eastern coast of Peninsular**												
Malaysia												
Tumpat, Kelantan Delta	8	1.3–2.0	45–60	114	11.1 ± 1.8	17.8 ± 1.7	49.1 ± 5.4	13.6 ± 1.6	0	0.3 ± 0.5	0.1 ± 0.4	121.8 ± 11.0
(1)		(8)	(2)	(1)	(9–13, 8)	(15–20, 8)	(40–57, 8)	(11–16, 8)	(0–0, 8)	(0–1, 8)	(0–1, 8)	(107–137, 8)
Setiu Lagoon, Terengganu	13	1.2–2.0	54–70	121–132	7.8 ± 3.0	16.1 ± 1.7	51.3 ± 5.9	13.7 ± 2.0	0	0.2 ± 0.4	0 ± 0	116.1 ± 11.4
(2)		(13)	(4)	(4)	(3–13, 13)	(13–18, 13)	(39–58, 13)	(11–17, 13)	(0–0, 13)	(0–1, 13)	(0–0, 13)	(94–136, 13)
Kuala Ibai, Terengganu	2	0.7–1.1	15		6.0	17.0	42	12	0	0	0	106
(3)		(2)	(1)		(6–6, 2)	(16–18, 2)	(42–42, 1)	(12–12, 2)	(0–0, 2)	(0–0, 2)	(0–0, 2)	(106–106, 1)
Pooled data from all of 3 sites		0.7–2.0	15–70	114–132	8.8 ± 3.0	16.7 ± 1.8	50.1 ± 5.8	13.5 ± 1.8	0	0.2 ± 0.4	0.04 ± 0.2	117.7 ± 11.4
		(23)	(7)	(5)	(3–13, 23)	(13–20, 23)	(39–58, 22)	(11–17, 23)	(0–0, 23)	(0–1, 23)	(0–1, 23)	(94–137, 22)
Singapore												
Nine sites in northern and southern coasts (Lee and Glasby 2015)^7^	54				9.0 ± 3.4	17.3 ± 2.5	49.2 ± 7.2	14.1 ± 2.5	0	0.1 ± 0.3	1.2 ± 2.1	120.1 ± 13.9
					(0–17, 54)	(11–23, 54)	(35–63, 54)	(10–22, 54)	(0–0, 53)	(0–1, 53)	(0–8, 51)	(93–148, 50)
Myanmar (Burma)												
Maungmagan (Lee and Glasby 2015)^7, 8^	8				5.8 ± 3.9	13.1 ± 2.0	41.3 ± 9.7	14.0 ± 2.9	0	0	0	101.3 ± 19.9
					(2–14, 8)	(11–17, 8)	(30–60, 8)	(11–20, 8)	(0–0, 8)	(0–0, 8)	(0–0, 8)	(80–138, 8)
India												
Water Lakes, St. 2, Calcutta (Lee and Glasby 2015)^7, 9^	1				10	12	38	7	0	1	2	90
					(1)	(1)	(1)	(1)	(1)	(1)	(1)	(1)
Near Calcutta (original description by Southern (1921)	1		88	123	10	(10–13)	50	(10–12)	0	(0–1)	up to 7	
			(1)	(1)								

Note: 1) Corresponding to site numbers in Fig. 1.; 2) Range and (number of available data); 3) Data from complete specimens; 4) Mean ± SD (range, and number of available data); 5) Larger value at a left or right side; 6) All total with numbers from both sides of groups II, IV, and VI; 7) Calculated based on the individual data shown in table 3 in Lee and Glasby (2015); 8) A part of syntypes of *Ceratonereisburmensis* Monro, 1937; 9) One of probable syntypes of Nereis (Nereis) glandicincta Southern, 1921.

Parapodia of first two chaetigers uniramous, all following parapodia biramous. Uniramous parapodia of first two chaetigers are without notoacicula. In subsequent biramous parapodia, notopodia consists of dorsal cirrus, dorsal ligule, prechaetal lobe and ventral ligule throughout (Fig. 4B–D); all ligules/lobes are conical with tapering tip throughout; ventral ligule subequal to or slightly smaller than dorsal ligule; prechaetal lobe much shorter than two ligules. Dorsal cirri tapering, shorter than notopodial dorsal ligule throughout (about half length). Glandular patches present along dorsal edge of dorsal and ventral ligules.

Neuropodia consisting of superior lobe, inferior (acicular) lobe, postchaetal lobe, ventral ligule and ventral cirrus present in anterior and middle body, but lack superior lobe in posterior body (Fig. 4C, D); postchaetal lobe present throughout; all ligules/lobes are conical with tapering tip throughout. Ventral cirrus is slender with tapering tip. Glandular patches present along ventral edge of neuropodial ligule/lobes.

Notochaetae all homogomph spinigers having long blades with finely serrated edges (Fig. 4E). Upper neurochaetae include homogomph spinigers with long blades (posteriorly) and heterogomph spinigers with short blades (anteriorly, Fig. 4F) throughout; some or most of heterogomph spinigers are replaced by heterogomph falcigers with slender blades (Fig. 4G) in middle body. Lower neurochaetae include heterogomph spinigers with long blades (posteriorly) and heterogomph spinigers with short blades (anteriorly) throughout; some or most of heterogomph spinigers with short blades are replaced by heterogomph falcigers in middle body. Heterogomph falcigers first appear around chaetiger 20 in both upper and lower neurochaetae.

###### Reproduction.

The coelom of a female specimen collected from Tumpat, Kelantan Delta on 13 August 2009 (BW 1.7 mm) was filled with oocytes (probably immature eggs) 100–140 µm in diameter.

###### Habitat.

Intertidal sandy or muddy flats in estuaries. Salinity in habitats highly varied; the salinity of surface water at Muara Kuala Setiu in Setiu Lagoon varied in a range from 22.4 to 28.3 psu (Nicholas 2018), while the salinity of interstitial water at the other sites, Setiu Lagoon, Kuala Ibai, and Tumpat, was in the range from 3.0 (Site 103 in Setiu Lagoon) to 16.5 psu (Tumpat).

###### Geographical distribution.

India, Myanmar, western Singapore, the eastern coast of Peninsular Malaysia. Based on synonymy with *C.burmensis*, and Southern (1921), Lee and Glasby (2015) and the present study.

## Discussion and conclusion

In the present study, an estuarine nereidid species, *Neanthesglandicincta* (Southern, 1921) is newly recorded at the eastern coast of Peninsular Malaysia in the South China Sea. The morphological characteristics of the present specimens which were collected from three estuaries in Malaysia well agreed with those of *N.glandicincta* originally described by Southern (1921) based on Indian specimens, and also those redescribed by Lee and Glasby (2015) based on Indian, Myanmar and Singapore specimens. The number of paragnaths in all groups on proboscis of our Malaysian specimens was within the range of the variation of *N.glandicincta* shown in the original description and redescription (Table 1). Therefore, our specimens can be clearly identified as *N.glandicincta*.

According to Atlas of Living Australia (2017), this species was previously recorded from Blue Lagoon which is situated near Port Dickson on the western coast of Peninsular Malaysia, based on specimen(s) deposited in the Northern Territory Museum and Art Gallery, Darwin, Australia (NTM W19065) (Chris Glasby, pers. comm.), though its taxonomic description has not yet been published. This record should be re-examined to clarify whether *N.wilsonchani* or other morphologically similar species are included or not.

Lee and Glasby (2015) showed that *Ceratonereisburmensis* Monro, 1937 (type locality: Maungmagan in Myanmar and off Bombay in India) is a junior synonym of *Neanthesglandicincta* (Southern, 1921), and that *N.glandicincta* was distributed in western Singapore, whereas the closely similar species, *N.wilsonchani* Lee & Glasby, 2015 was distributed in eastern Singapore. In the present study on three estuaries in eastern Malaysia, we could not find *N.wilsonchani* in spite of geographical proximity between our sampling sites and the type locality of *N.wilsonchani* (eastern Singapore). The record of *C.burmensis* from Jeram, Selangor, western peninsular Malaysia by Polgar et al. (2015), is likely to represent one of these two species, but this needs to be verified.

Lee and Glasby (2015) described the epitokous metamorphosis of both *N.glandicincta* and *N.wilsonchani* based on sexually mature males and females collected from Singapore in a period from December to April. However, we were not able to collect any epitokous specimens of *N.glandicincta* probably because our sampling period was limited to August.

Previous records of “*N.glandicincta*” and “*Ceratonereisburmensis*” on the coasts of South China Sea (Khlebovich 1963, Wu 1967, Wu et al. 1985) and Australia (Rullier 1965) should be re-examined because some morphologically similar but distinct species (*N.wilsonchani* or other cryptic species) may have been confused with *N.glandicincta* or *C.burmensis.*Lee and Glasby (2015) and Sato (2017) suggested that another cryptic species similar to *N.glandicincta* and *N.wilsonchani* may be distributed on the coasts of South China Sea and East China Sea.

## Supplementary Material

XML Treatment for Neanthes

XML Treatment for Neanthes glandicincta
